# Immunologic burden links periodontitis to acute coronary syndrome: levels of CD4 + and CD8 + T cells in gingival granulation tissue

**DOI:** 10.1007/s00784-023-05448-7

**Published:** 2024-03-07

**Authors:** Nezahat Arzu Kayar, İlhami Çelik, Muammer Gözlü, Kemal Üstün, Mihtikar Gürsel, Nilgün Özlem Alptekin

**Affiliations:** 1 Department of Periodontology, Faculty of Dentistry, Akdeniz University, Antalya, 07058 Turkey; 2 Department of Biochemistry, Faculty of Veterinary Medicine, Selcuk University, Konya, Turkey; 3 Dentesthetic Oral and Dental Clinic, Konya, Turkey; 4 Department of Periodontology, Faculty of Dentistry, Bezmialem University, Istanbul, Turkey; 5 Department of Periodontology, Faculty of Dentistry, Başkent University, Ankara, Turkey

**Keywords:** Acute coronary syndrome, Macrophage, CD4 +, CD8 +, Gingival tissue

## Abstract

**Objective:**

To investigate the proportional variation of macrophage and T-lymphocytes subpopulations in acute coronary syndrome (ACS) patients, its association with periodontitis (P), and to compare with control individuals.

**Subjects and methods:**

Three groups of subjects participated: one group consisted of 17 ACS patients with P (ACS + P), another group consisted of 22 no ACS + P patients, and a control group consisted of 23 participants with gingivitis (no ACS + G). Macrophage, CD4 + , and CD8 + T-lymphocytes and CD4 + /CD8 + ratio values in gingival tissue were determined histometrically.

**Results:**

Significant differences were found among three groups regarding the mean number of macrophage (no ACS + P > ACS + P > no ACS + G; *p* < 0.05) and CD8 + T-lymphocytes (no ACS + P > ACS + P > no ACS + G; *p* < 0.05). Significant variations were observed between the groups both CD4 + T-lymphocytes densities (ACS + P > no ACS + P and ACS + P > no ACS + G; *p* < 0.05) and CD4 + / CD8 + ratio (no ACS + P < no ACS + G and ACS + P < no ACS + G; *p* < 0.05).

**Conclusions:**

The increased number of CD8 + T-lymphocytes in both group ACS + P and group no ACS + P resulted in a reduction of the CD4 + /CD8 + ratio in gingival tissue when compared with no ACS + G group.

**Clinical relevance:**

The decrease of CD4 + /CD8 + ratio in gingival tissue reflects periodontitis and may be associated with severe adverse outcomes in people with ACS.

## Introduction

Periodontitis is a chronic inflammatory disease caused by microorganisms and host response to these microorganisms in disease susceptible individuals. Putative periodontal pathogens, such as *Aggregatibacter actinomycetemcomitans*, *Porphyromonas gingivalis*, *Prevotella tannerella forsythia*, *Campylobacter rectus*, and *Fusobacterium nucleatum*, form complexes that virulently interact with each other [1]. Efficient and controlled innate and adaptive immune responses at this site are important for the maintenance of immune homeostasis [2]. If innate immune responses (neutrophils and macrophages which possess very effective but nonspecific microbe clearance machineries) fail to eradicate the infection, then it activates the effector cells of adaptive immune responses (lymphocytes with highly selective and specific microbe killing capabilities) [3, 4]. In this second stage of the immune response, CD4 + T helper/inducer lymphocytes (T_h/i_) and CD8 + cytotoxic/suppressor T lymphocytes (T_c/s_), which are some of the cellular immune effector cells, play a central role in immunoregulation as they respond to nonspecific inflammatory reactions but also to humoral immune responses [5, 6]. A net increase in the number of macrophages in tissues has been demonstrated in periodontitis lesions as compared to gingivitis lesions [7]. A reduced CD4 + /CD8 + (T_h/i_/T_c/s_) ratio in the periodontal lesion [8–10] and the crevice [11] has described as well as a correlation between the number of T cells and the degree of periodontal inflammation [11].

The most severe serious cardiovascular disease seen in the clinic is an acute coronary syndrome (ACS), including unstable angina (UA) and acute myocardial infarction (AMI); the development of ACS is sudden, and the disease progresses very rapidly and has a high mortality rate. The main pathological basis of ACS is atherosclerosis (AS), which is considered a chronic inflammatory condition [12]. The evidence seems to suggest that by eliciting both the innate and adaptive components of host immunity to modified lipoproteins, AS exhibits several features of immune-mediated inflammatory disease [13, 14] Monocytes/macrophages play important roles in the various stages of atherosclerosis, the development of complications from the very beginning, and then in ACS during myocardial healing and remodeling [15]. ACS is characterized by a sharp increase in the effector T-cell numbers, unlike a decrease in regulatory T-cell numbers [16]. Disruption of adaptive immunity, involving mainly the CD4 + T_h/i_ lymphocytes, was identified in patients with ACS and systemic inflammation [17, 18].

The immunological burden of periodontitis has recently been linked with the acute coronary syndrome [19]. Multiple periodontal pathogens have been identified in atherosclerotic plaques [20]. Periodontal infections can contribute to atherosclerosis and thromboembolic events by repeatedly challenge bacterial LPSs and proinflammatory cytokines to the vascular endothelium and arterial wall. Soluble form of the urokinase plasminogen activator receptor (suPAR) levels may reflect endothelial dysfunction and inflammation, potentially influencing immune responses and tissue damage in both periodontitis [21, 22] and cardiovascular disease [23, 24]. It has been demonstrated that the presence of periodontitis, through a sustained inflammatory burden on gingival tissues, can lead to increased levels of both local and systemic suPAR, which may constitute an additional adverse risk factor for endothelial dysfunctions and cardiovascular diseases development [25].

Epithelial cells have the ability to recognize the Pattern Recognition Receptors (PRRs) on immune cells related to microbial pathogens, cellular stress, and damage-associated molecular patterns. These cells can release cytokines and chemokines and can even influence neutrophils’ adhesion and differentiation when interacting with bacteria. This emphasizes the crucial role epithelial cells have in the immune response. There are three main categories of PRRs: Toll-like receptors (TLRs), RIG-I-like receptors, and Nod-like receptors (NLRs). Recent research suggests that NLRs contribute to forming inflammasomes, which are protein complexes involved in triggering inflammation [26]. Recent studies have shown that activation of Nucleotide Binding Oligomerization Domain, Leucine Rich Repeat and Pyrin Domain Containing Protein 3 (NLRP3) contributes to local and systemic inflammation in both periodontitis [27, 28] and acute coronary syndrome (ACS) [29, 30]. 

In patients with macrophage phenotype, vascular monocytes and macrophages create a high inflammatory response by directly accelerating atherosclerosis and thromboembolic events [31]. The role of lymphocyte function in the relationship between ACS and periodontitis is less defined. Hence, gingival tissue investigation might have the ability to provide a new perspective on the immune activation of coronary artery disease. In addition to being a localized inflammatory disease, periodontitis is also associated with modifications in the quantity and kind of circulating inflammatory cells. Periodontitis as a whole sets off a distinctive systemic inflammatory cell profile that may exacerbate or even cause other systemic disorders to develop [32]. In light of this knowledge, we aimed to evaluate the level of differentiation of the immune system in relation to T-lymphocytes in ACS patients and its association with their periodontal disease status, to explore CD4 + Th/i and CD8 + Tc/s and macrophage concentrations in the gingival tissue samples of healthy individuals with periodontitis (no ACS + P), healthy individuals with gingivitis (no ACS + G), and ACS patients with periodontitis (ACS + P).

## Materials and methods

### Study population

This research was approved by the human subjects ethics board of the Ethics Committee of Selcuk University Faculty of Dentistry (project number = 2008/3–1) and was conducted by the Helsinki Declaration of 1975, as revised in 2013.

This study included 17 patients with ACS and periodontitis (ACS + P group), 22 patients without ACS and with periodontitis (no ACS + P group), and 23 patients with gingivitis (no ACS + G group).

Inclusion criteria for groups were as follows:

- *ACS* + *P group:* Patients with clinical signs and symptoms of myocardial ischemia including unstable angina (UA), non-ST-segment myocardial infarction elevation (NSTEMI), and ST-segment myocardial infarction elevation (STEMI) are hospitalized in the Department of Cardiology at Selcuk University, Meram Medical Faculty. Those patients had also a diagnosis of periodontitis stages II or III grade B [33] (age ranged 25–57 years, 2 female and 15 male).
- *No ACS* + *P group:* Patients without ACS and with periodontitis stages II or III grade B (age ranged 30–60 years, 8 female and 14 male) were otherwise systemically healthy.
- *No ACS* + *G group:* Patients with gingivitis was used as reference (age ranged 23–52 years, 10 female and 13 male)

**Fig. 1:**
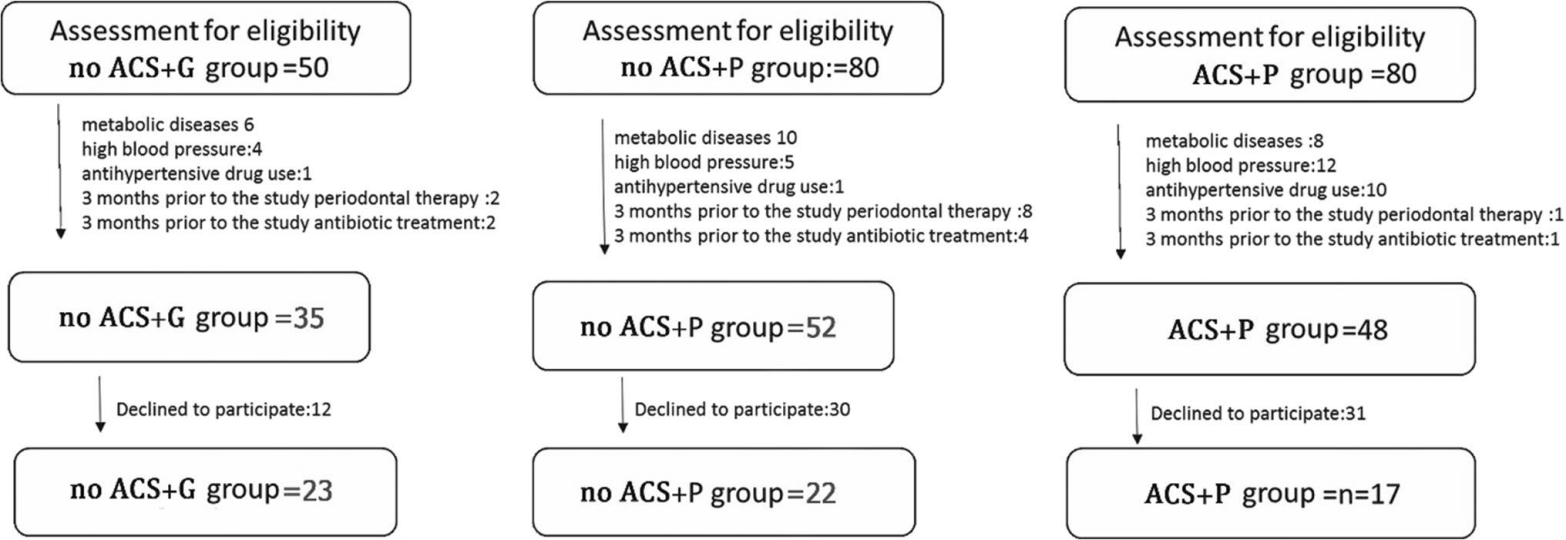
Flow chart of the inclusion and exclusion of participants

Exclusions for both groups included metabolic diseases, high blood pressure, antihypertensive drug use, and 3 months before the study, periodontal therapy, and antibiotic treatment. Figure 1 displays a flowchart of the investigation method for research. The study was registered at Thai Clinical Trials.gov (TCTR20200518001).

### Clinical periodontal evaluation

As in a previous study [34], each ACS + P participant received a comprehensive periodontal examination approximately 1 month after care and hospital evacuation. Periodontal health status of patients was evaluated by plaque index (PI) [35], papilla bleeding index (PBI) [36], probing depth (PD), and clinical attachment loss (CAL). All probing scores were measured with Williams periodontal probe calibrated in millimeters. Clinical attachment loss was determined relative to the cementoenamel junction.

### Gingival tissue biopsy

At baseline, the clinical periodontal parameters were not significantly different between CP and CVD groups in biopsy-sampling sites (Mann–Whitney *U* test, *p* > 0.05). During subgingival curettage, granulation tissue biopsies (3 × 3 mm in size) from the interdental sulcus region with gingival index 2, pocket depth > 5 mm, and BOP, including gingival epithelium and ligament, were obtained after clinical periodontal evaluation.

The gums around the teeth of 23 patients who underwent third molar tooth extraction were used in the no ACS + G group. The biopsies were characterized by the following criteria from clinically healthy sites: no loss of attachment (CAL < 3 mm), PD 3 mm negative, and BOP negative.

### Tissue sampling, processing, and histologic evaluation

Tissue sampling, processing, and demonstration of alpha-naphthyl acetate esterase (ANAE) enzyme, which is specific for T lymphocytes and macrophages with different staining patterns, and immunohistochemical staining of CD4 + and CD8 + lymphocytes were performed as described by Kayar et al. (2020). The specimens were observed under a light microscope at high magnification (X1000). The lymphocytes with reddish-brown cytoplasmic granules were scored as ANAE + . Macrophages were distinguished with a diffuse fine granular cytoplasmic staining. In immunohistochemically stained specimens, the cells having lymphocyte morphology and displaying brownish membrane HRP positivity were considered as positive for the primary antibody (CD4 + or CD8 +) according to what antibody was used. In each specimen, ANAE + lymphocytes and macrophages and CD4 + and CD8 + cells were counted in the unit area (283,490.27 square micrometer), and the mean cell concentration values of the groups were expressed as cell count/unit area (CC/UA).

### Statistical analysis

In order to characterize the participants of this study, descriptive analysis of the data was carried out and then comparisons were made between their mean CD4 + and CD8 + T lymphocyte levels. To compare the mean levels of CD4 + and CD8 + T lymphocytes, the CD4 + /CD8 + ratio among all groups, the Kruskal–Wallis test was used. The Mann–Whitney *U* test was performed for comparisons between pairs. The Bonferroni test was used for multiple comparison correction. *P* values 0.016 were considered statistically significant.

## Results

A total of 62 patients participated in the study, 42 men (67.7%) and 20 women (32.3%), with a mean age of 44.50 ± 8.36 years old. There were no significant differences in age, sex, and educational level distribution among the study groups (*P* > 0.05). Low-dose aspirin and various combinations of nitrates, beta-blockers, and/or calcium antagonists have been treated the patients with ACS + P. Table 1 describes serum cholesterol, triglyceride, HDL, LDL, fibrinogen, and CRP levels in Group ACS + P and Group no ACS + P.

**Table 1:** Serum cholesterol, trigliseride, HDL, LDL, fibrinogen, and CRP levels in the periodontitis (P) and the acute coronary syndrome (ACS) with periodontitis groups

	P (*n* = 22)	ACS (*n* = 17)
Cholesterol (mg/dl)	184.83 ± 39.59 181.5 101–281	190.38 ± 55.98 178 104–265
Trigliseride (mg/dl)	153.05 ± 90.40 138 59–374	161.15 ± 139.74 109 75–592
HDL (mg/dl)	39.35 ± 8.30 39 26–57	40.15 ± 11.00 36 27–59
LDL (mg/dl)	112.47 ± 37.90 112 55–176	118.07 ± 47.42 110 51–186
Fibrinogen (mg/dl)	321.62 ± 91.72 292.50 222–500	399.05 ± 96.33 389.25 272–563
CRP (mg/dl)	4.72 ± 2.62 4 1.70–11.70	6.06 ± 2.86 5.60 1.30–9.50

No significant differences were found between the ACS with periodontitis group and the P group in any of blood markers (Mann–Whitney *U* test, *p* > 0.05)

The PI, GI, BOP, PD, and CAL among groups with periodontitis (ACS + P and no ACS + P groups) were similar in Table 2 (*p* > 0.05). In Group no ACS + P, the rate of ANAE-positive lymphocytes and macrophage concentrations were significantly (*p* < 0.05) higher than that of the ACS + P group (Table 3).

**Table 2:** Clinical periodontal parameters in the periodontitis (P) and the acute coronary syndrome with periodontitis (ACS) groups

	P (*n*= 22)	ACS (*n*= 17)
PI	2.61 ± 0.49 3.00 2.00–3.00	2.42 ± 0.51 2.00 2.00–3.00
PBI	2.42 ± 0.50 2.00 2.00–3.00	2.35 ± 0.49 2.00 2.00–3.00
PD (mm)	5.47 ± 0.74 5.00 5.00–7.00	5.20 ± 0.56 5.00 5.00–7.00
CAL (mm)	5.76 ± 1.13 5.00 5.00–9.00	5.61 ± 0.76 5.00 5.00–7.00

The clinical periodontal parameters were not significantly different between the ACS group with periodontitis and the P group in biopsy-sampling sites (Mann–Whitney *U* test, *p* > 0.05)

**Table 3:** ANAE + cells, macrophages, T lymphocytes, CD4 + T lymphocytes, CD8 + T lymphocytes and CD4 + /CD8 + ratio in the healthy (H), the periodontitis (P), and the acute coronary syndrome with peritonitis (ACS) groups

	H (*N*= 23)	P (*N*= 22)	ACS (*N*= 17)
ANAE + cells	32.13 ± 10.30 32 15–52	88.50 ± 25.63^*^ 82.50 56–134	42.11 ± 21.92^**#**^ 36 22–55
Macrophage	7.43 ± 3.05 7 3–5	29.68 ± 14.94^*^ 30 9–74	11.64 ± 4.18^* #^ 10 6–21
T lymphocyte	24.69 ± 10.21 22 7–42	58.81 ± 24.87^*^ 48.5 27–107	30.47 ± 20.36^**#**^ 25 7–100
CD4 + T lymphocyte	15.73 ± 7.24 13 4–28	28.04 ± 14.05^*^ 22 12–51	15.11 ± 10.93^**#**^ 13 4–54
CD8 + T lymphocyte	8.95 ± 3.69 10 3–15	30.40 ± 10.45^*^ 28 15–52	15. 05 ± 9.66^* #^ 14 3–46
CD4/CD8	1.85 ± 0.61 1.80 0.55–2.75	0.91 ± 0.36^*^ 0.81 0.56–2.00	1.05 ± 0.28^*^ 1.07 0.61–1.50

All of the parameters were significantly different between the H, the P, and the ACS with peritonitis groups (Kruskal–Wallis test, *p* < 0.05)

^*^Significantly different from the H group (Bonferroni adjusted Mann–Whitney *U* test, *p* < 0.05)

^#^Significantly different from the P group (Bonferroni adjusted Mann–Whitney *U* test, *p* < 0.05

**Fig. 2:**
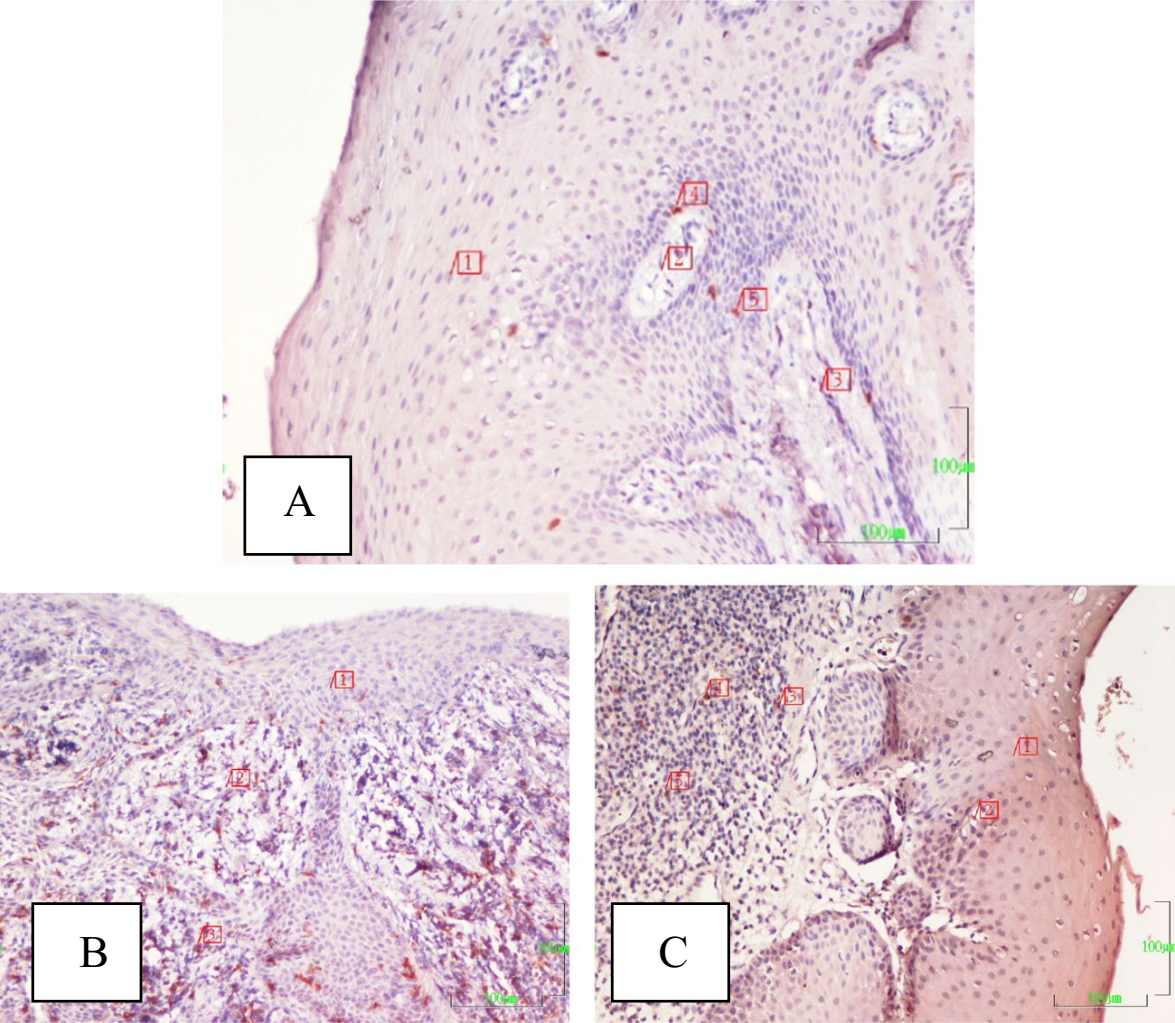
**A**–**C** CD4 + T-lymphocyte populations are seen in the sections from healthy (no ACS + G) group (**A**, 1-epithelium, 2-peg, 3 and 5-CD4 + T-lymphocytes), periodontitis (no ACS + P) group (**B**, 1-epithelium, 2-peg, 3-CD4 + T-lymphocyte), and acute coronary syndrome (ACS + P) group (**C**, 1- epithelium, 2-peg, 3–5-CD4 + T-lymphocytes) group are seen. The section of the P group is the most intensively populated with CD4 + positive T-lymphocytes. CD4 immunohistochemical staining. Magnification bar: 100 µm

**Fig. 3:**
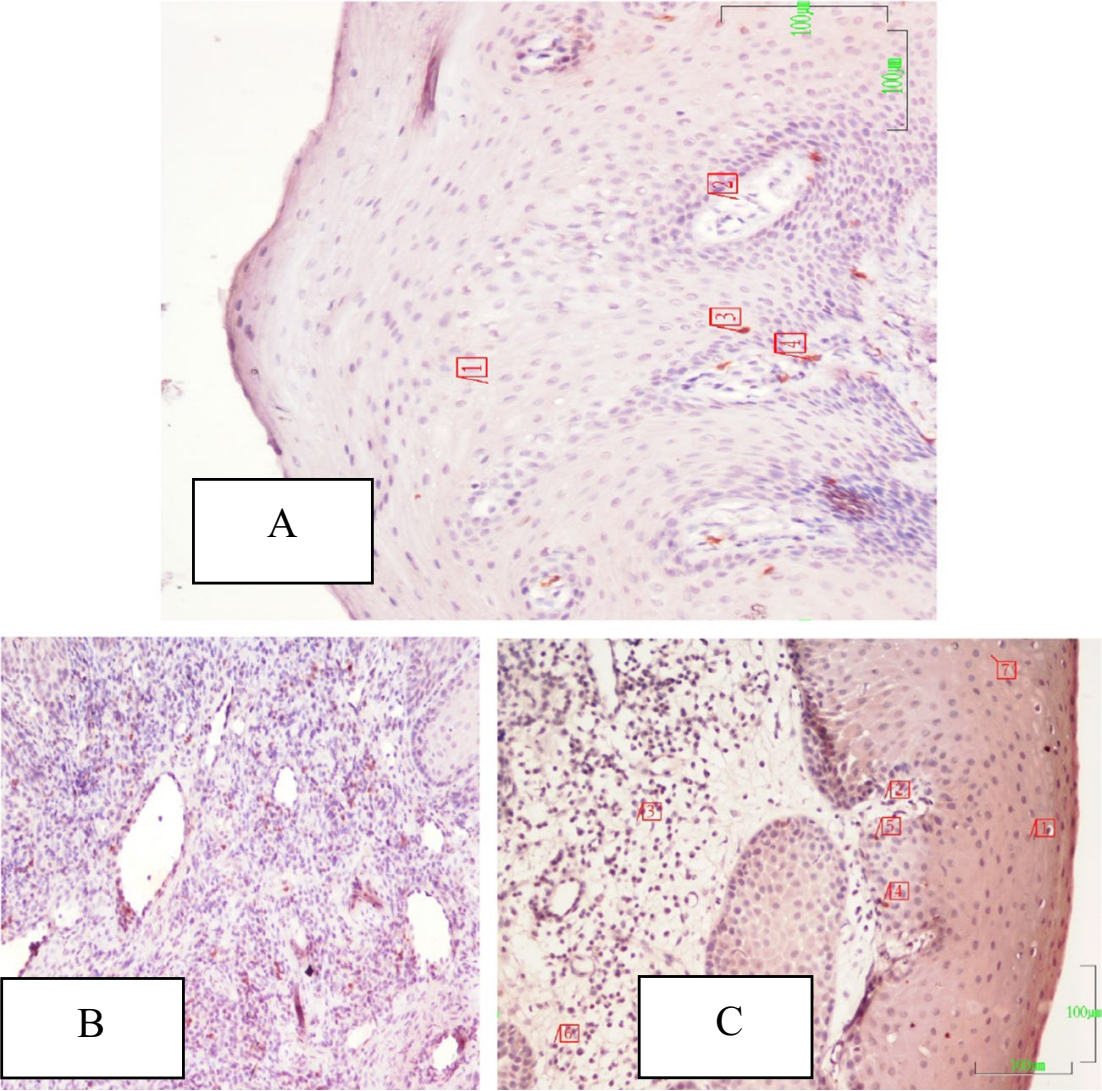
**A**–**C** CD8 + T-lymphocyte populations are seen in the sections from no ACS + G (**A**, 1- and 7 epithelium, 2-peg, 3 and 4 CD4 + T-lymphocytes), periodontitis (**B**) (no ACS + P) group, and acute coronary syndrome (ACS + P) group (**C**, 1-epithelium, 2-peg, 3 and 6-CD + negative lymphocytes, 4 and 5-CD8 + T-lymphocytes) are seen. In the section of the P group, CD4 + positive T-lymphocyte population (brownish stained cells) is more intensive. CD8 + immunohistochemical staining. Magnification bar: 100 µm

The lowest ANAE-positive lymphocyte level was found in the no ACS + G group (*p* < 0.05). Mean T lymphocyte concentration in the no ACS + P group was higher than those of the other groups (*p* < 0.05). CD4 + T-lymphocytes in the no ACS + P group was higher than that of the control and ACS + P groups (Fig. 2). The CD4 + /CD8 + ratio in the no ACS + G group was higher than those of the other groups (*p* < 0.05). CD8 + T lymphocyte and macrophage concentrations in the no ACS + P group were significantly higher (*p* < 0.05) than those of the no ACS + G and ACS + P groups (Fig. 3).

## Discussion

The results of this study yielded that CD4 + T-lymphocytes in the no ACS + P group were higher than that of the control and ACS + P groups (*p* < 0.05). Also, CD8 + T lymphocyte and macrophage concentrations in the no ACS + P group were significantly higher than those of the no ACS + G and ACS + P groups (*p* < 0.05). Another result of our study was that the CD4 + /CD8 + ratio in the no ACS + G group was higher than those of the other groups (*p* < 0.05). The numbers of ANAE + cells and T-lymphocytes significantly increased in the no ACS + P group compared to the ACS + P and no ACS + G groups (*p* < 0.05). 

Periodontitis is well known to play a crucial role in both initiation and propagation ACS. In this study, we analyzed the immune response of periodontitis patients with/without ACS in periodontal tissues and compared patients with gingivitis. Patients with an abnormally exuberant inflammatory response often have a hyper inflammatory monocyte and macrophage phenotype and risk for coronary heart disease as well as destructive periodontal disease [31]. We found higher macrophage levels in the no ACS + P and ACS + P groups than the no ACS + G group. It was noteworthy that the macrophage level was higher in the no ACS + P group than the ACS + P group. This result may have caused aspirin therapy to inhibit LPS-induced activation of macrophages [37].

The proportion of CD8 + T-lymphocytes in the gingival tissue was higher in the ACS + P group than in the no ACS + G group. It is found that CD8 + T lymphocytes increased in inflammatory gingival tissues [10], crevicular fluid [11], and/or peripheral blood [38]. CD8 + T-lymphocytes have been suggested to extend humoral and cellular responses in vivo through the production of chemokines at the peripheral site of the infection [39]. Moreover, it was found that depleting CD8 + cells in animal models by using monoclonal antibodies decreases atherosclerosis, as well as reducing apoptotic cells and nucleic necrotic damage [40]. These findings could be explained by the presence of a higher number of CD8 + T-lymphocytes in group ACS + P relative to group H in the current study. Since the total number of CD4 + T-lymphocytes in the ACS + P group showed approximately the same values as in the no ACS + G group, the decrease in the CD4 + /CD8 + ratio in the ACS + P group compared to the no ACS + G group.

If T-lymphocytes, such as those present in atherosclerotic plaques, are exposed to persistent antigens for long periods, this may lead to differentiation of lymphocyte populations that could be expressed in a decreased adaptive response. This high degree of differentiation is however not only found in CD4 + T cells but also in CD8 + T cells [41]. We found in our study that the ACS + P group which used aspirin had significantly lower CD4 + and CD8 + numbers than the P group. As an anti-inflammatory agent, aspirin mainly exerts its pharmacological effect by inhibiting COX enzymes which lead to reduced synthesis of pro-inflammatory PGs, including PGE_2_ [42]. Aspirin directly suppresses T cell activation or proliferation and/or inhibits T cell-mediated adaptive immune response-related cytokine production [43]. It is well known that aspirin significantly decreases the percentage and number of CD4 + T-lymphocytes in vitro [44–46]. Similarly, all of the ACS patients in the present study, who received aspirin (mostly 100 mg /daily) treatment at least for 1 month, had significantly lower CD4 + and CD8 + numbers than the no ACS + P group. In our study, when ACS + P and no ACS + P groups with the same severity of periodontal disease were compared, it was defined that the amount of CD4 + T-lymphocytes in the gingival tissue in the ACS + P group receiving aspirin treatment decreased sharply and reached similar levels with the no ACS + G group. Regular use of aspirin has also been related to low levels of CD8 + T-lymphocyte [47]. We found that the amount of CD8 + T-lymphocytes in the gingival tissue decreased significantly compared to the no ACS + P group in the ACS + P group undergoing aspirin therapy, but was still significantly higher than the no ACS + G group. It is the first study to indicate that CD8 + T-lymphocytes could be more resistant than CD4 + T-lymphocytes to the inhibiting effects of the aspirin therapeutic dosage in gingival tissue.

The CD4 + /CD8 + ratio is decreased by the presence of periodontal pathogens in atherosclerotic lesions [48]. In our study, we found a significant decrease in the CD4 + /CD8 + ratio in gingival tissue with periodontitis (ACS + P group and no ACS + P group) compared to the no ACS + G group, quite similar to a previous research [8–11]. It is noteworthy that while we observed a decrease in CD4 + and CD8 + T lymphocyte amounts in gingival granulation tissue due to the use of aspirin in the ACS + P group relative to the no ACS + P group, the CD4 + /CD8 + ratio was similar. This may be a result of the gingival tissue region sampled in both groups has similar clinical periodontal parameters. There has been a renewed interest in the utility of the CD4 + /CD8 + ratio as a strong predictor of immune activation and immune senescence [49]. Consequently, the predictive value of CD4 + /CD8 + ratio in gingival tissue during the follow-up of patients with periodontitis as an independent risk factor for ACS + P needs future prospective studies involving more patients.

NLRP3, a key component of the innate immune system, has been implicated in the inflammatory processes underlying both periodontitis [28–30] and ACS [27, 28]. It has been found that LPS in *P. gingivalis* could activate NLRP3 inflammasome and inhibit the function of mesenchymal stem cells (MSCs), thereby accelerating MSC dysfunction and delaying wound healing [50]. It was reported that low-concentration aspirin could inhibit the formation and activation of NLRP3 inflammasome [51]. In a recent study, Vandanmagsar et al. demonstrated that the suppression of NLRP3 reduces inflammation, increases the number of naive T cells, and decreases the number of effector T cells such as CD4 and CD8 cells in adipose tissue in an animal model [52]. Similarly, in this study, CD4 and CD8 cell counts in the ACS + P group were lower than the no ACS + P group. This result may be due to the fact that the patients were on low-dose aspirin treatment.

Within the limits of this study, despite the similar periodontal disease severity, a decrease in CD4 + and CD8 + T lymphocyte levels was observed in ACS + P patients compared to P patients. For decades, aspirin has been commonly used as a non-steroidal anti-inflammatory medication that can modulate conditions such as cardiovascular disease, cancer, and periodontal disease [40, 53]. According to the results of our study, although aspirin use reduced macrophage, CD4 + T-lymphocyte, and CD8 + T-lymphocytes amount in the ACS + P group, it did not clinically affect periodontal disease severity (similar to BOP) and CD4 + / CD8 + ratio.

The first limitation of this study is the relatively small sample size. Secondly, this was a cross-sectional study, and the results are descriptive. The relationship between adaptive immune changes and ACS is exploratory as it is not possible to reveal a causal link. The aspect of causality in the acute phase can only be explained by Mendel randomization or intervention studies. Therefore, long-term follow-up of patients with periodontitis by prospective studies may explain the causal relationship. Additionally, the inclusion of suPAR in this study, which systematically reflects ongoing local inflammation [54], which has currently proven to be a valuable prognostic biomarker of periodontitis and CHD [25], would have been useful in elucidating the link between ACS and periodontitis. Because it predicts future disease development, measuring suPAR provides an opportunity to take action with targeted interventions in those at the highest risk for adverse health outcomes [54]. 

## Conclusions

In summary, these results suggest that an increase in the number of CD8 + T-lymphocytes in ACS + P patients will disrupt the peripheral immune system balance and cause inflammation, resulting in the emergence of pathological ACS processes. Our findings also show that periodontitis is associated with an impaired adaptive immune response not only in CD4 + T-cells but also in CD8 + T lymphocytes, with a high degree of differentiation and, therefore, excessive functional responsiveness. This T cell-mediated adaptive immunity imbalance may play a key role in periodontitis, potentially increasing the risk of ACS.

## Data Availability

The data used to support the findings of this study are available from the corresponding author upon reasonable request.
